# Ultra-high gain diffusion-driven organic transistor

**DOI:** 10.1038/ncomms10550

**Published:** 2016-02-01

**Authors:** Fabrizio Torricelli, Luigi Colalongo, Daniele Raiteri, Zsolt Miklós Kovács-Vajna, Eugenio Cantatore

**Affiliations:** 1 Department of Information Engineering, University of Brescia, via Branze 38, Brescia 25123, Italy; 2 Department of Electrical Engineering, Eindhoven University of Technology, Groene Loper 19, PO Box 513, Eindhoven 5600MB, The Netherlands

## Abstract

Emerging large-area technologies based on organic transistors are enabling the fabrication of low-cost flexible circuits, smart sensors and biomedical devices. High-gain transistors are essential for the development of large-scale circuit integration, high-sensitivity sensors and signal amplification in sensing systems. Unfortunately, organic field-effect transistors show limited gain, usually of the order of tens, because of the large contact resistance and channel-length modulation. Here we show a new organic field-effect transistor architecture with a gain larger than 700. This is the highest gain ever reported for organic field-effect transistors. In the proposed organic field-effect transistor, the charge injection and extraction at the metal–semiconductor contacts are driven by the charge diffusion. The ideal conditions of ohmic contacts with negligible contact resistance and flat current saturation are demonstrated. The approach is general and can be extended to any thin-film technology opening unprecedented opportunities for the development of high-performance flexible electronics.

## Abstract (web summary)

Organic field-effect transistors offer limited gain due to the large contact resistance and the channel length modulation. Here, Torricelli *et al.* show a new transistor architecture where the charge injection and extraction are driven by the charge diffusion and a gain larger than 700 is achieved.

## Graphical abstract

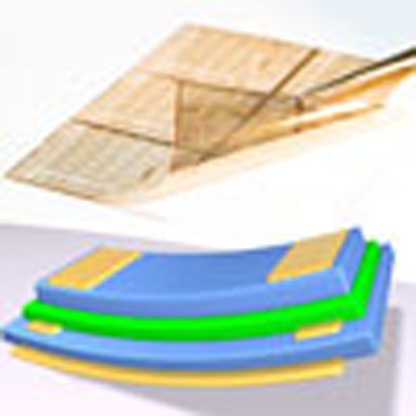

Transistors fabricated with organic, polymeric, amorphous-oxide and carbon-based materials are the basis of emerging technologies for the development of lightweight, large-area and flexible electronics^1^,^2^,^3^,^4^,^5^,^6^. Large-area electronics manufactured at near-to-room temperature on plastic foils aims at enabling new applications where mechanical flexibility, integration in wrapping materials and ultra-low cost are paramount. To fabricate a transistor in flexible technologies, nanometre-thick layers of metals, insulators and semiconductor are stacked together and the semiconductor is directly contacted with the metal electrodes. The overall transistor performance intimately depends on three physical processes: the charge injection from the source electrode to the semiconductor, the charge transport through the semiconductor and the charge extraction at the drain electrode. The impressive development of high-mobility semiconductors^7^,^8^,^9^ and short channel-length transistors^10^,^11^ urgently demand high-quality contacts and proper transistor design^12^,^13^. Unfortunately, the energetic matching between abruptly contacted metal–semiconductor materials is challenging, especially at near-to-room temperature^13^. Electrons and holes must overcome large energy barriers to flow from a material to the other, resulting in a large contact resistance, large device-to-device variations and low transistor amplification^13^,^14^,^15^,^16^,^17^,^18^.

The figure-of-merit that determines the intrinsic amplification of a transistor is the gain=*g*_m_/*g*_o_, where *g*_m_=∂*I*_D_/∂*V*_G_ is the transconductance and *g*_o_=∂*I*_D_/∂*V*_D_ is the output conductance. High-gain transistors are essential for the development of large-scale and robust circuits, high-sensitivity sensors, and adequate signal amplification in sensing systems. Unfortunately, organic field-effect transistors (OFETs) typically show a gain of the order of tens^17^,^18^. The low gain measured in OFETs is due to the large contact resistance that results in a small *g*_m_ and to the channel length modulation that results in a large *g*_o_. Therefore, high-gain OFETs need, at the same time, both high-quality contacts and flat current saturation.

Ohmic contacts with small contact resistance require efficient charge injection and extraction. In organic electronics, the contact optimization is performed on a case-by-case basis, depending on the semiconductor, electrodes and device architecture. Despite *ad-hoc* approaches^18^,^19^,^20^,^21^,^22^,^23^,^24^,^25^ such as doping, surface treatments and materials blending enable to reduce the contact resistance, a general and simple method is desirable. In addition, the channel length modulation dependents on the specific OFET architecture and geometries, which determine how the charge carriers are extracted at the drain^17^,^18^.

Here we show a new organic transistor with high-quality contacts and flat current saturation. Thanks to the charge diffusion triggered by the transistor architecture, the charge carriers are efficiently injected and extracted from the contacts to the channel, independently of the energy barrier at the contacts. As a prototype and remarkable example, we fabricate Diffusion-driven Organic Field-Effect Transistors (named DOFETs) on flexible plastic substrates with an industrial thin-film technology. The theoretical and experimental analysis unambiguously show that the diffusion-driven contact, proposed in this work for the first time, is fundamental to dramatically improve the charge injection and extraction in organic thin-film field-effect transistors. The ideal conditions of negligible contact resistance and fully flat current saturation are demonstrated. These conditions maximize together the transconductance and the output resistance of the transistors, resulting in OFETs with exceptionally high gain (>700).

## Results

### Structure and electrical characteristics of the transistor

**Figure 1:**
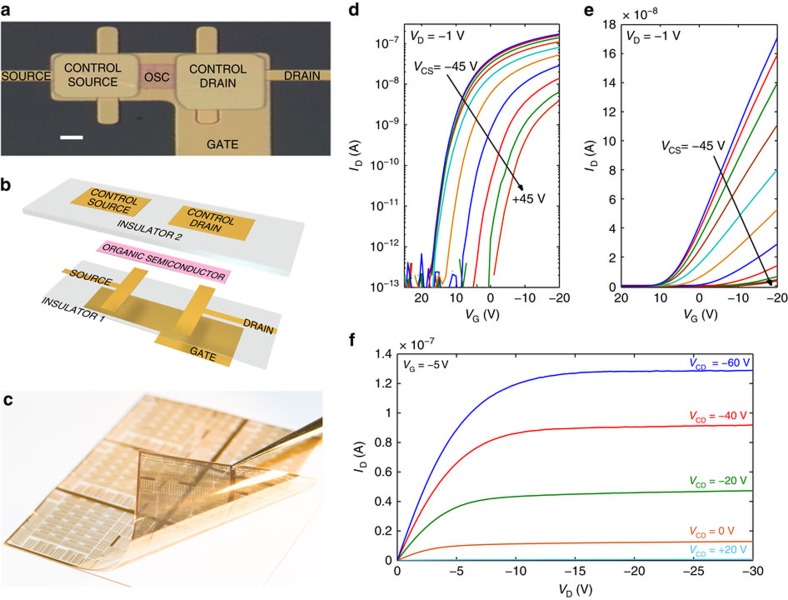
Transistor architecture and characteristics. (**a**) Top-view optical image of a diffusion-driven organic field-effect transistor (DOFET) fabricated on plastic foil OSC is the organic semiconductor. Scale bar, 5 μm. (**b**) DOFET components. Photolithographically patterned gold is used for metal electrodes (named gate, source, drain, control source, control drain), the insulators (insulators 1 and 2) are photoimageable polymers (polyvinylphenol), and the organic semiconductor is a solution-processed pentacene. The material thicknesses are detailed in the Supplementary Fig. 1. (**c**) Photograph of the plastic (PEN) foil with the measured transistors detached from the glass substrate. The transistors are fabricated with an industrial thin-film technology with three metal layers. (**d**,**e**) Measured transfer characteristics at several control source voltages. The *V*_CS_ step is 10 V, *V*_S_=0 V and *V*_CD_=0 V. The DOFET channel width and length are *W*=100 μm and *L*=12.5 μm, respectively. (**f**) Measured output characteristics at several control drain voltages.

The top-view image and the three-dimensional structure of the diffusion-driven organic transistor are shown in Fig. 1a,b. The transistors are bottom-gate co-planar where the gate is patterned first by using photolithography. Thereafter, we deposited by spin coating a photoimageable polymer (polyvinylphenol) used as a gate insulator (named insulator 1) followed by gold source (S) and drain (D) electrodes patterned by a lift-off process. A 100-nm-thick film of pentacene is deposited by spin coating and patterned. A thick layer of polyvinylphenol (named insulator 2) is deposited by spin coating and used as insulator and capping layer. Finally, two electrodes named ‘control source' (CS) and ‘control drain' (CD) are patterned on the top of the insulator 2 in front of the source and drain electrodes. The transistors are fabricated on a plastic polyethylene naphthalate foil (Fig. 1c) and the overall process temperature is lower than 150 °C. Further details on the transistors fabrication and geometries are shown in Supplementary Fig. 1. The measured transfer and output curves are shown in Fig. 1d–f and Supplementary Fig. 2.

### Operation of the transistor

**Figure 2:**
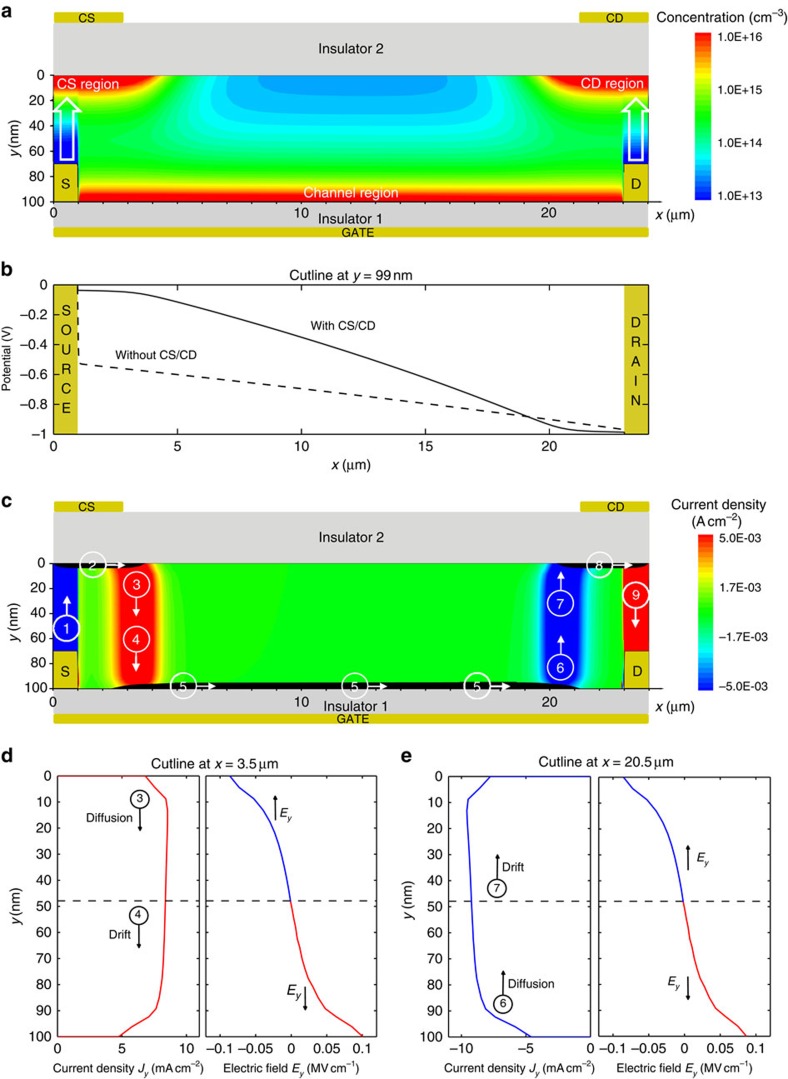
DOFET operation. Two-dimensional numerical simulations. The applied voltages are *V*_G_=−5.1 V, *V*_S_=0 V, *V*_D_=−1 V, *V*_CS_=−60 V, *V*_CD_=−60 V. Geometrical and physical parameters are listed in the Supplementary Fig. 1 and in the Methods section, respectively. (**a**) Charge concentration in the organic semiconductor. The white arrows depict the charge injection from the source and drain electrodes into the semiconductor when the control source and control drain electrodes are biased. The *x*-to-*y* scale ratio is 1:200. (**b**) Quasi-Fermi potential at *y*=99 nm with (full line) and without (dashed line) CS/CD. Without CS/CD about half of *V*_DS_ drops at the source and it is required for the charge injection. (**c**) Current density: *x*-component *J*_*X*_ (black area) is equal to 1 A cm^−2^, and the *y*-component *J*_Y_ is shown with colour scale levels. (**d**) Current density *J*_*Y*_ and electric field *E*_*Y*_ along the *y*-direction at *x*=3.5 μm. In the range *y*=[0–47] nm, the current is driven by the diffusion, and in the range *y*=[47–100] nm, the current is driven by the drift. (**e**) *J*_*Y*_ and *E*_*Y*_ along the *y*-direction at *x*=20.5 μm. In the range *y*=[0–47] nm, the current is driven by the drift, and in the range *y*=[47–100] nm, the current is driven by the diffusion.

The DOFET operates as follow. An appropriate voltage applied to CS and CD, creates a vertical electric field orthogonal to the S/D contact surface. It triggers a charge injection from the upper surface of S/D into the semiconductor (Fig. 2a). In equilibrium (*V*_S_=*V*_D_=0 V, no current flows), the electric-field below CS/CD is counterbalanced by the injected-charges that are accumulated in the semiconductor region below CS/CD. When a source-drain voltage is applied (|*V*_DS_|>0), the charge carriers flow from source to drain despite the contact energy barriers and the potential drop at the contacts is negligible (Fig. 2b).

More in detail (Fig. 2c), the charge carriers accumulated in the CS region move to the right-edge of the CS region itself, attracted by the drain potential (Fig. 2c, arrow 2). As a consequence, the vertical electric field at the left-hand-side of the CS region is not shielded anymore and, despite the energy barrier, other charges can be injected by the source electrode (arrow 1). The excess of the charge carriers at the right-hand-side of the CS region are pushed to the bottom channel by the diffusion against the vertical electric field (arrow 3). As shown in Fig. 2d, few nanometres far from the CS region the vertical electric-field changes direction under the influence of the gate potential and the charge carriers are eventually pulled into the transistor channel (arrow 4). As a result, the CS region acts as an ideal source. The key physical mechanism triggered by the transistor architecture is the charge diffusion, which takes place in less than *L*_diff_=50 nm (Fig. 2d) when the semiconductor thickness is *t*_S_=100 nm. We also verified that the diffusion length scales accordingly with the semiconductor thickness (that is, *L*_diff_≅25 nm when *t*_S_=50 nm).

The charge carriers injected into the channel drift to the drain (Fig. 2c, arrow 5) under the force of the longitudinal electric field. When the charge carriers reach the right edge of the channel, they are blocked by the energy barrier at the drain contact, and the local concentration increases. The charges are no more counterbalanced by the gate electric field, and they can diffuse to the CD region (arrow 6) in correspondence of the CD region edge. As shown in Fig. 2e, few nanometres far from the channel the vertical electric field changes direction, the charge carriers are pulled into the CD region (arrow 7) and eventually diffuse (arrow 9) to the drain. The CD region acts as an ideal drain.

The idea is that in the DOFETs the charge injection and extraction do not take place directly from the source and drain metal electrodes as in conventional transistors but, instead, the charge carries are injected by the CS region and are extracted by the CD region. The injection and the extraction are driven by the diffusion triggered by the transistor architecture. As a result, when enough charge carriers are accumulated in the CS and CD regions, the charge injection and extraction are independent of the applied voltages (viz. *V*_CS_ and *V*_CD_) and the CS and CD regions behave like ideal source and drain for the transistor channel. Therefore, as confirmed by the two-dimensional (2D) numerical simulations shown in Supplementary Fig. 3, the gate electrode is not required to overlap the source and drain electrodes.

The potential at the insulator1–organic interface calculated by means of 2D numerical simulations is shown in Fig. 2b. In the DOFET, the potential drop at the contacts is negligible even if the energy barrier at the metal–semiconductor contacts is 0.5 eV, that is a typical barrier at the metal–organic contacts. In contrast, in a conventional organic transistor (viz. without CS and CD), the charge carriers must overcome the energy barrier flowing from the channel to the S/D electrodes and vice versa. Owing to the energy barrier, the channel is disconnected from the S/D electrodes and more than the half of the drain voltage drops at the contacts (Fig. 2b). The large contact resistance severely limits the transistor performances and this is even worse in case of high-mobility semiconductors and/or short-channel lengths.

### Experimental analysis

**Figure 3:**
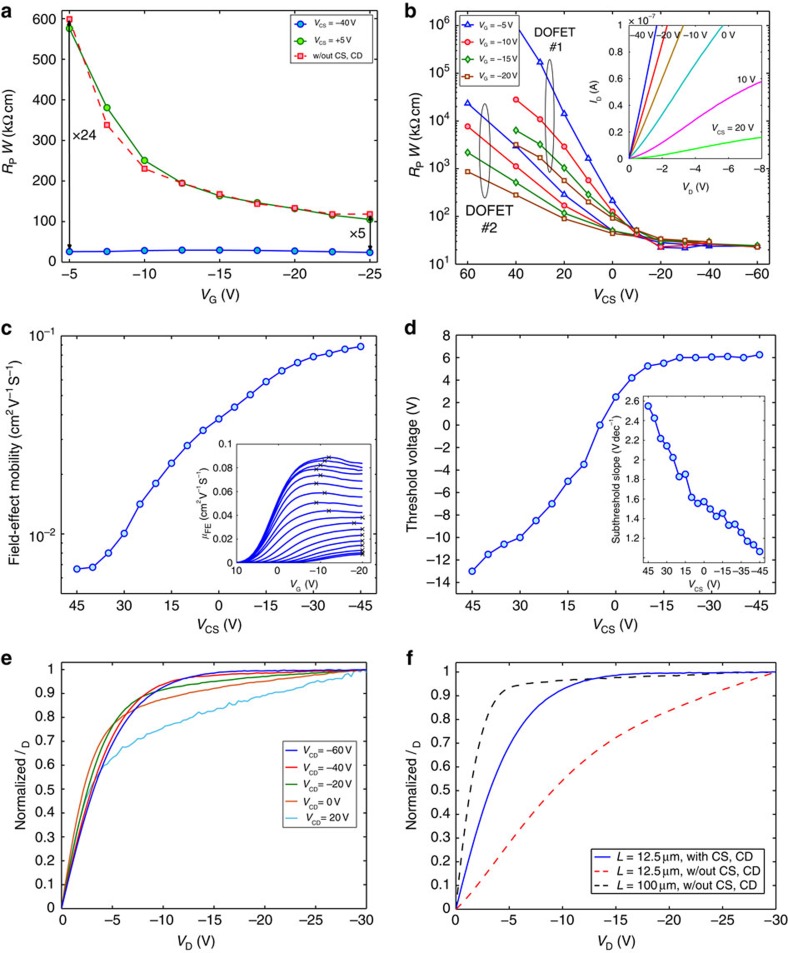
DOFET measurements and parameters. When it is not specified, the applied voltages are: *V*_S_=0 V, *V*_D_=−1 V, *V*_CS_=0 V, *V*_CD_=0 V, and the transistors geometries are: *W*=100 μm, *L*=12.5 μm. (**a**) Width-normalized contact resistance *R*_P_ as a function of the gate voltage *V*_G_. *R*_P_ is calculated with the method^26^. In the conventional OFET (viz. without CS and CD), *R*_P_ decreases with *V*_G_, whereas in the DOFET, *R*_P_ is independent of *V*_G_. When the control source is biased at *V*_CS_=+5 V, the DOFET works as a conventional coplanar OFET. (**b**) *R*_P_ vs *V*_CS_ at various *V*_G_ measured on two nominally identical DOFETs. When *V*_CS_<−10 V, *R*_P_ is the same for both the DOFETs and it is independent of both *V*_G_ and *V*_CS_. Inset: measured output characteristics of a DOFET at several *V*_CS_. (**c**) Maximum overall field-effect mobility vs *V*_CS_. The inset shows the field-effect mobility as a function of the gate voltage: *μ*_FE_=(*L*/*W*) (∂*I*_D_/∂*V*_G_)/(*C*_i_ *V*_D_). The × symbol is the maximum value of each curve. (**d**) Threshold voltage (*V*_TH_) as a function of *V*_CS_. *V*_TH_ is the intercept to the *V*_G_-axis of the *I*_D_ linear fit. Inset: Subthreshold slope as a function of *V*_CS_. (**e**) Normalized output characteristics of the DOFET measured at various *V*_CD_. *I*_D_ is normalized by its maximum value at *V*_D_=−30 V. In saturation, the DOFET is an ideal current generator because the current is diffusion driven. The most important short-channel effect due to the channel-length modulation vanishes. The *V*_CD_ controls the charge extraction at the drain electrode, which has a strong impact on the output conductance (*g*_O_=∂*I*_D_/∂*V*_D_). (**f**) Normalized output characteristics of a DOFET and two conventional OFETs (viz. without CS and CD).

The effectiveness of the proposed approach is further assessed by means of the experimental results shown in Fig. 3. Figure 3a shows the measured contact resistance *R*_P_ as a function of the gate voltage *V*_G_. The contact resistance of the DOFET biased at *V*_CS_=−40 V (that corresponds to an electric field |*E*_*Y*-VCS_|=0.28 MV cm^−1^) is equal to *R*_P[DOFET]_=20 kΩ cm, which is lower than the contact resistance in conventional OFETs with Au-pentacene-doped contacts^20^ and, more importantly, *R*_P[DOFET]_ is independent of *V*_G_. In contrast, the contact resistance of an organic transistor without CS/CD (conventional coplanar transistor) fabricated with the same materials and process is *V*_G_ dependent. It is up to 24 times larger than that of the DOFET and, even at large gate voltages (*V*_G_=−25 V, that is, |*E*_*Y*−VG_|=0.7 MV cm^−1^), *R*_P[OFET]_>5 × *R*_P[DOFET]_. Analogous results are obtained comparing the DOFET with a conventional staggered OFET (Supplementary Fig. 4).

To give more insight, Fig. 3b shows the *R*_P_-*V*_CS_ characteristic of two nominally identical DOFETs for several *V*_G_. *R*_P_ is controlled by *V*_CS_ despite the gate voltage. Indeed, at low gate voltage (*V*_G_=−5 V, that is, |*E*_*Y*-VG_|=0.14 MV cm^−1^), *V*_CS_ modulates *R*_P_ by more than four orders of magnitude, and at large *V*_G_=−20 V (|*E*_*Y*-VG_|=0.57 MV cm^−1^), *R*_P_ still depends on *V*_CS_. Interestingly, when *V*_CS_<−20 V (that is, |*E*_*Y*-VCS_|=0.14 MV cm^−1^) the contact resistance is negligible compared with the channel resistance (Supplementary Fig. 5) and it is independent of both *V*_G_ and *V*_CS_. As confirmed by the measurements shown in the inset of Fig. 3b, this is the experimental evidence that the current enhancement originates from the improved charge injection at the source. According to the physical insight obtained by means of the 2D numerical simulations, at *V*_CS_<−20 V, the accumulated CS-region is an ‘infinite' charge reservoir, the charge diffusion efficiently sustain the charge injection required by the channel, and the CS-region behaves like an ohmic contact. On the other hand, at *V*_CS_>+5 V, the diffusion-driven charge injection is turned-off, the contact resistance increases, the drain current lowers and it increases super-linearly with *V*_D_ as usually obtained in contact limited transistors^13^,^15^,^16^,^19^. We can conclude that it is possible to control (enhance or reduce) the charge injection at the source contact through nanometre-scale charge diffusion.

Comparing the *R*_P_ obtained for two nominally identical DOFETs (Fig. 3b), it results that when the virtual-ohmic source contact is not formed, the transistors show different *R*_P_, whereas as soon as the virtual-ohmic source contact is formed (*V*_CS_<−20 V), *R*_P_ becomes the same for both the DOFETs. According to refs 10, 12, 13, these measurements suggest that the metal–organic contact is a source of variability. As the DOFET suppresses the contact resistance, the variability due to *R*_P_ is reduced as well. This feature is essential for the large-scale integration of flexible circuits. Moreover, the improved charge injection results in a larger overall field-effect mobility (Fig. 3c) as well as in a reduced threshold voltage (Fig. 3d) and steeper subthreshold slope (inset Fig. 3d). Figure 3c shows that the maximum mobility of a DOFET with *L*_[DOFET]_=12.5 μm is close to 0.1 cm^2^ V^−1^ s^−1^ and it corresponds to the mobility measured in long-channel OFETs (*L*_[OFET]_=100 μm), where the contact resistance is negligible. Figure 3d shows that by means of *V*_CS_ the DOFET can be turned into a multi-threshold transistor and the improved DOFET (*V*_CS_<−20 V) operates in depletion-mode. In unipolar technologies, depletion-mode transistors are essential to design high-performance circuits^27^,^28^ and the electrical control of the threshold voltage is extremely important to improve the circuit robustness^27^,^28^,^29^.

**Figure 4:**
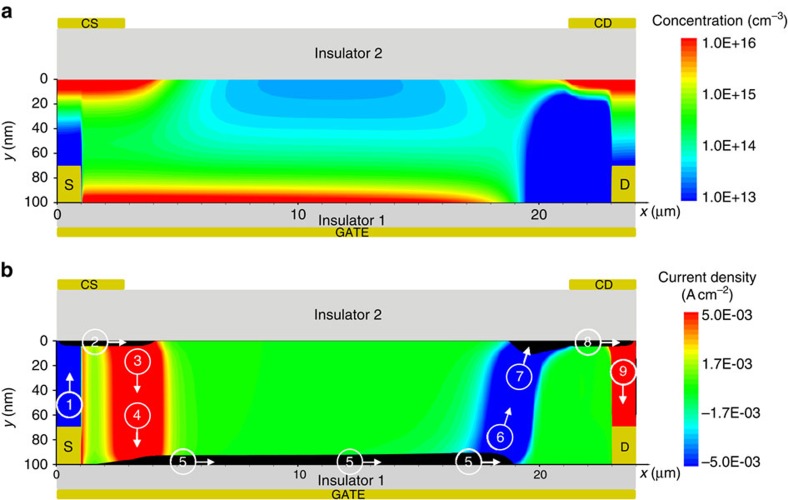
2D numerical simulations of a DOFET operating in saturation. The applied voltages are *V*_G_=−5.1 V, *V*_S_=0 V, *V*_D_=−10 V, *V*_CS_=−60 V, *V*_CD_=−60 V. (**a**) Charge concentration in the organic semiconductor. (**b**) Current density: *x*-component *J*_*X*_ (black area) is equal to 1 A cm^−2^, and the *y*-component *J*_*Y*_ is shown with colour scale levels. For the sake of clarity, the positions of control source (CS), control drain (CD) and gate electrodes are shown. Geometrical and physical parameters are listed in the Supplementary Fig. 1.

When the transistor operates in linear region, the energy barrier at the drain side of the channel is smaller than that at the source side. On the other hand, in saturation (|*V*_G_|<|*V*_D_|), a wider energy barrier is present at the drain, independently of the metal/semiconductor properties (Fig. 4a). Therefore, we investigated the impact of the control drain in saturation. The output characteristics (*I*_D_–*V*_D_) of the DOFET measured at various *V*_CD_ are shown in Fig. 1f. As expected, *I*_D_ increases with *V*_CD_ and, more importantly, at large (negative) *V*_CD_ the DOFET shows fully flat current saturation. The impact of *V*_CD_ on the current saturation is readily visible in Fig. 3e where the *I*_D_–*V*_D_ characteristics are normalized with respect to the maximum *I*_D_ measured at *V*_D_=−30 V. At *V*_CD_<−40 V, the detrimental effect of the channel length modulation on the drain current is completely suppressed and the DOFET behaves like an ideal current generator.

This can be explained in the light of the previous analysis. In saturation, the charge carriers drift to the right-edge of the channel (pinch-off region), and diffuse to the CD region (Fig. 4b, arrow 6). Few nanometres far from the channel edge, the vertical electric-field changes direction because of the control drain voltage and, in turn, the charge carriers are pulled into the CD region (arrow 7). Now, the excess charges are no more in equilibrium with the vertical electric-field and can diffuse to the drain (arrow 9). As the charge-extraction from the accumulated layer (viz. CD region) is diffusion driven, the drain current is independent of the drain voltage as far as *V*_CD_ is greater than *V*_D_.

Figure 3f shows the comparison between a DOFET with a channel length *L*_[DOFET]_=12.5 μm (full line), and two conventional coplanar OFETs with *L*_[OFET1]_=12.5 μm (red dashed line) and *L*_[OFET2]_=100 μm (black dashed line). Interestingly, the channel length modulation of the DOFET biased at *V*_CD_=−60 V is completely suppressed: it is even smaller than that of the long-channel OFET2. This is also more evident when the DOFET is compared with a conventional staggered OFET (Supplementary Fig. 6) where the channel length modulation is very large because the source and drain electrodes are placed at the opposite side of the gate. These results confirm that *V*_CD_ controls channel length modulation and in turn the output resistance of the DOFET. The channel length modulation is one of the most important short-channel effects and it limits the transistor amplification.

**Figure 5:**
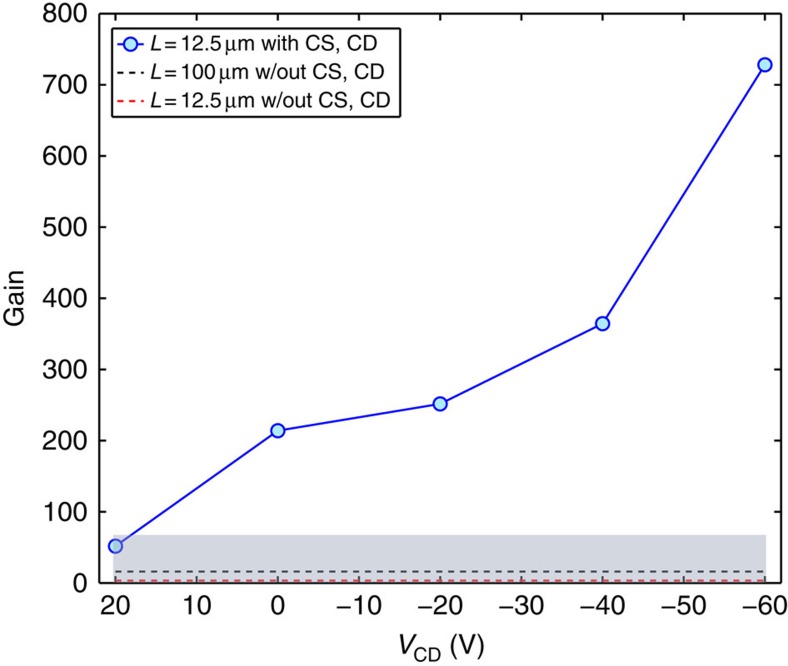
DOFET gain. Measured gain as a function of *V*_CD_. The applied voltages are *V*_G_=−5 V, *V*_S_=0 V, *V*_CS_=−20 V. The transistors width is *W*=100 μm. The DOFET (full line with symbols) length is *L*=12.5 μm. The OFET lengths are *L*=12.5 μm (red dashed line) and *L*=100 μm (black dashed line). The other geometries are the same. The DOFET and OFET are fabricated with the same materials (Supplementary Fig. 1). The grey area shows the gain obtained in OFETs^11^,^16^,^17^,^18^,^30^,^31^,^32^,^33^,^34^,^35^.

Figure 5 shows the maximum gain measured in a DOFET as a function of *V*_CD_ (full line with symbols). According to Figs 1f and 3e, the gain depends on *V*_CD_ because it controls both the contact resistance at the drain and the channel length modulation. When *V*_CD_=−60 V, the gain is larger than 700. This is the largest gain ever reported for OFETs. It is one order of magnitude larger than the gain usually obtained in OFETs^11^,^16^,^17^,^18^,^30^,^31^,^32^,^33^,^34^,^35^.

## Discussion

The ultra-high gain measured in the DOFET is achieved thanks to the diffusion-driven charge injection and extraction. In particular, when the CS and CD regions are accumulated, they act as ideal contacts for the channel and the diffusion enables the efficient and voltage-independent charge injection and extraction. In the DOFET, the CS and CD regions are at the opposite side of the channel and resemble a staggered OFET with ideal ohmic contacts. It is important to note that in the DOFET this condition is always achieved, thanks to the accumulated CS and CD regions. The charge flow from/to the CS/CD regions and the channel is driven by the charge diffusion, and thus the contact resistance is independent of the gate (Fig. 3a) and drain (inset Fig. 3b) voltages, the saturation current is independent of *V*_D_, and an ultra-high gain is obtained.

As a comparison, the gain measured in the conventional OFET1 (*L*_[OFET1]_=12.5 μm, red dashed line) and OFET2 (*L*_[OFET2]_=100 μm, black dashed line) are shown in Fig. 5. As expected in both cases, the gain is much lower than that measured in the DOFET at any *V*_CD_ because in the OFET1 the current is contact limited and the channel modulation is large, whereas in the OFET2 the contact resistance is negligible but the channel length is large and hence *g*_m_ is small. In OFETs, the contact resistance can be reduced by means of the contact engineering and optimization^18^,^19^,^20^,^21^,^22^,^23^,^24^,^25^, and the proper choice of the transistor architecture^36^,^37^. Indeed, staggered OFETs are more tolerant to the contact resistance with respect to the coplanar OFETs because in the staggered transistors the contact area (of the order of microns) is larger than that of coplanar transistors (of the order of nanometres). On the other hand, in staggered transistors the source and drain electrodes are at the opposite side of the channel and, when operated in saturation, the channel length modulation is larger than that in coplanar OFETs. As an alternative approach, the split-gate OFETs^33^,^34^ are based on a coplanar architecture and lower the contact resistance thanks to the gate bias-assisted charge injection^38^. However, the channel length modulation is not suppressed because the secondary gates are coplanar with the source and drain electrodes, the charge extraction is not diffusion driven and, as a result, the gain is comparable with that typically measured in OFETs (of the order of tens).

**Figure 6:**
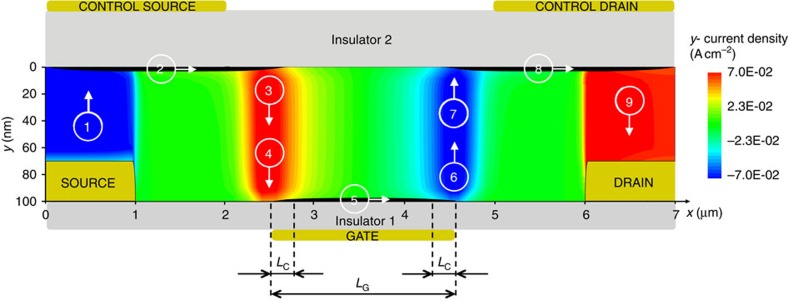
2D numerical simulations of a DOFET with minimized capacitances. Current density: *x*-component *J*_*X*_ (black area) is 10 A cm^−2^, and the *y*-component *J*_*Y*_ is shown with colour scale levels. For the sake of clarity, the positions of control source (CS), control drain (CD) and gate electrodes are shown. Geometrical and physical parameters are listed in the Supplementary Fig. 1. The applied voltages are *V*_G_=−5.1 V, *V*_S_=0 V, *V*_D_=−1 V, *V*_CS_=−60 V, *V*_CD_=−60 V.

In addition to the high-gain, another advantage offered by the DOFET is the possibility to maximize the charge injection/extraction area at the source and drain electrodes, whereas minimizing the overlap between the gate and the electrodes. The 2D numerical simulations in Supplementary Fig. 3 and Fig. 6 show that the gate is not required to overlap the source and drain electrodes because the charge injection/extraction takes place from/to the CS/CD accumulated regions. At the same time, the CS and CD electrodes can be overlapped (without the drawback of extra capacitance) with the source and drain electrodes in order to exploit the full area of the electrodes that is typically in the range 5–10 μm (in our DOFET it is 5 μm). Thanks to the charge diffusion, taking place at the edge of the accumulated CS and CD regions, also the overlap between the gate and the CS and CD electrodes is not required. Moreover, the numerical simulations in Fig. 6 show that the equivalent contact length where the charges are injected/extracted is only *L*_C_=0.25 μm, which is suitable for the megahertz operation^11^.

Finally, it is worth noting that the voltages required to form the charge-accumulated CS and CD regions are independent of the DOFET operation. For example, by setting *V*_CS_=*V*_CD_=−40 V, the DOFET operates as a conventional OFET with ideal ohmic contacts and ultra-high gain. Therefore, the two control electrodes can be connected together and the external circuit design and lines required for the proposed transistor structure is the same of that required for dual-gate transistors. The latter have been successfully used to fabricate an organic microprocessor with 3,381 dual-gate OFETs^39^. Moreover, an alternative approach is to replace the CS and CD electrodes with fixed charges trapped into the insulator 2 (ref. 40). Another very interesting approach would be the replacement of both the CS and CD electrodes and the insulator 2 with electric dipoles (Supplementary Fig. 7) by local molecular self-assembly functionalization^41^,^42^ of the top surface of the organic semiconductor in front of the source and drain electrodes.

In summary, the DOFET shows that it is possible to dramatically enhance the charge injection and extraction at the metal/semiconductor contacts by means of the nanometre-scale charge diffusion. The enhanced charge injection allowed us to reduce the threshold voltage by more than 15 V, and to increase the field-effect mobility about ten times, approaching the organic semiconductor transport limit also in short-channel transistors. The enhanced charge extraction enables the complete suppression of the channel-length modulation. We show that a short-channel DOFET behaves like an ideal current generator: its channel-length modulation is even smaller than that of an eight times longer organic transistor fabricated in the same technology. These features lead to the fabrication of high performance organic transistors with a unique benefits combination: negligible contact resistance, small device-to-device variability, and exceptionally high gain (>700).

Thanks to the transistor here proposed we theoretically explain and experimentally demonstrate for the first time that the charge diffusion can play a crucial role in organic transistors. Moreover, the ability to independently enhance or reduce the charge injection, transport and extraction in organic semiconductors makes the DOFET the ideal test-bed to study the fundamental physical processes taking place in organic semiconductors and at the metal–organic interfaces.

The proposed approach is a universal method to obtain high-quality contacts without the need of materials or process optimizations. Moreover, according to the approach proposed in ref. 43, the DOFET combined with ambipolar semiconductors could be used to electrically enhance the charge injection of one charge type and to suppress the other. This feature is very relevant for the low-cost fabrication of high-gain and low-power ambipolar complementary electronics.

The diffusion-driven organic transistor opens up new opportunities for the large-scale integration of flexible electronics, high-sensitivity sensors and ultra-large signal amplification in sensing systems.

## Methods

### Two-dimensional numerical simulations

The coupled drift–diffusion, Poisson and current continuity equations are solved together^43^,^44^,^45^. The simulation parameters are the following: relative permittivity of semiconductor *ɛ*_rs_=3, relative permittivity of insulators (1 and 2) *ɛ*_ri_=3.757, highest occupied molecular orbital (HOMO) energy level *E*_HOMO_=2.8 eV, lowest unoccupied molecular orbital (LUMO) energy level *E*_LUMO_=5.2 eV, effective density of HOMO states *N*_HOMO_=10^21^ cm^−3^, effective density of LUMO states *N*_LUMO_=10^21^ cm^−3^, holes effective mobility *μ*_h_=0.1 cm^2^ V^−1^ s^−1^, electrons effective mobility *μ*_e_=0.1 cm^2^ V^−1^ s^−1^, metal electrodes work function *Φ*_m_=4.7 eV (the hole energy barrier at the source/drain metal-semiconductor is *Φ*_B_=0.5 eV), Schottky barrier lowering Δ*Φ*_B_=*e* [*e E*/(4 *π ɛ*_0_ *ɛ*_rs_)]^(1/2), where *e* is the elementary charge, *E* is the electric field and *ɛ*_0_ is the vacuum permittivity.

## Additional information

**How to cite this article**: Torricelli, F. *et al.* Ultra-high gain diffusion-driven organic transistor. *Nat. Commun.* 7:10550 doi: 10.1038/ncomms10550 (2016).

## Supplementary Material

Supplementary Information Supplementary Figures 1-7 and Supplementary References
