# Supplementary material for: Ultra-high gain diffusion-driven organic transistor
Source: Nat Commun. 2016 Feb 1;7:10550. doi: 10.1038/ncomms10550 (PMC4740436; doi:10.1038/ncomms10550)
Supplement: Supplementary Information — Supplementary Figures 1-7 and Supplementary References [file ncomms10550-s1.pdf]

# Supplementary Figures

a

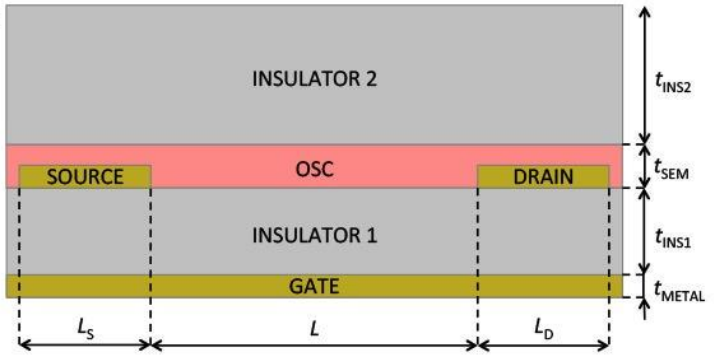


b

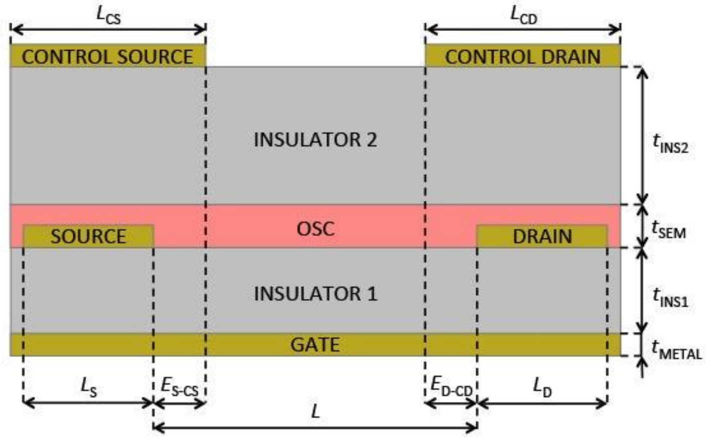


c

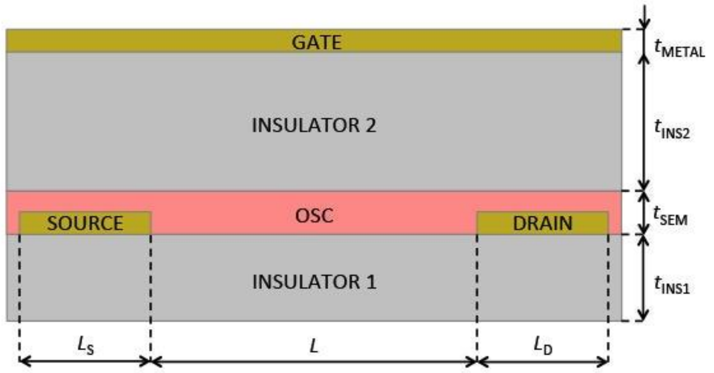


**Supplementary Figure 1 | Transistors geometries.** All the transistors (i.e. coplanar OFETs, DOFETs, and staggerd OFETs) are fabricated with the same materials and fabrication process, on the same plastic foil. For all transistors the metal electrodes thickness is $t_{METAL}$ = 30 nm, the insulator 1 thickness is $t_{INS1}$ = 350 nm (insulator 1 capacitance per unit area is $C_i$ = 9.5×10$^{-9}$ F cm$^{-2}$), the semiconductor thickness is $t_{SEM}$ = 100 nm, and the insulator 2 thickness is $t_{INS2}$ = 1400 nm (insulator 2 capacitance per unit area is $C_{i(2)}$ = 0.25×$C_i$ = 2.375×10$^{-9}$ F cm$^{-2}$). (**a**) Conventional bottom-gate bottom-contact coplanar OFET. The "insulator 2" is used as a capping layer. The OFET geometries are the following: source length $L_S$ = 5 μm, drain length $L_D$ = 5 μm, channel width $W$ = 100 μm, and channel length $L$ = 12.5 μm (named OFET1) or $L$ = 100 μm (named OFET2). (**b**) DOFET architecture. The DOFET geometries are the following: source length $L_S$ = 5 μm, drain length $L_D$ = 5 μm, channel width $W$ = 100 μm, channel length $L$ = 12.5 μm, control source length $L_{CS}$ = 10 μm, control drain length $L_{CD}$ = 10 μm, control source to source enclosure $E_{S\text{-}CS}$ = 5 μm, and control drain to drain enclosure $E_{D\text{-}CD}$ = 5 μm. (**c**) Conventional bottom-contact top-gate staggered DOFET. The staggered DOFET geometries are the following: source length $L_S$ = 5 μm, drain length $L_D$ = 5 μm, channel width $W$ = 100 μm, and channel length $L$ = 12.5 μm.

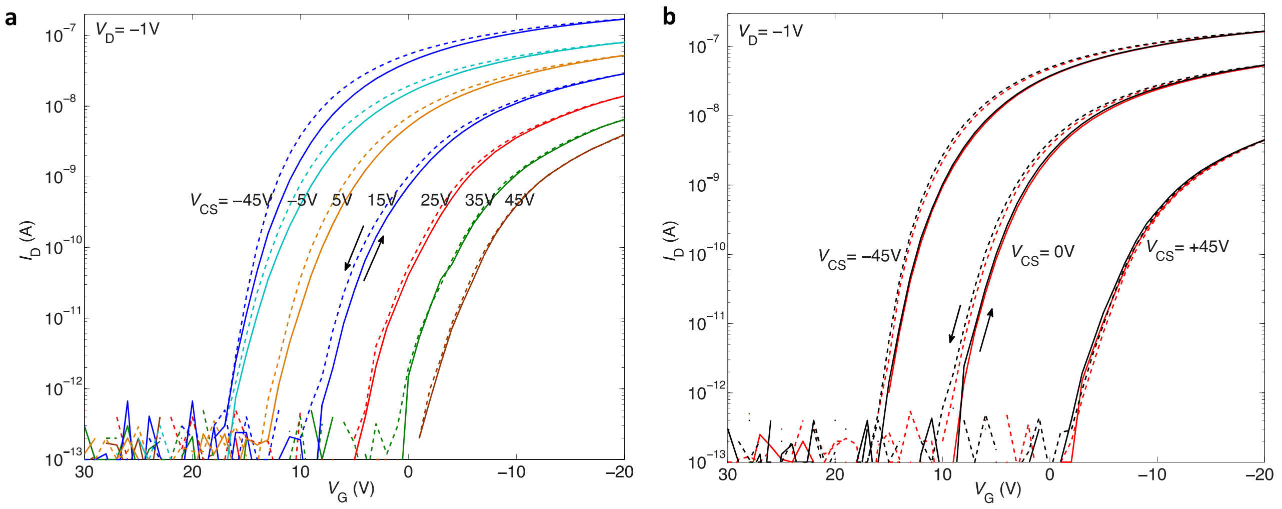


**Supplementary Figure 2 | DOFET bias stress stability.** Measured current-voltage transfer characteristics. (**a**) The DOFET is biased at $V_D$ = -1 V, $V_{CD}$ = 0 V, and $I_D$-$V_G$ is measured at several $V_{CS}$. (**b**) The $I_D$-$V_G$ is measured at several $V_{CS}$. This is repeated two times. The black and red lines are the two set of measuments. The time required for each single measurement (forward and backward) is 120 s and the time elapsed between the two set of measurements (black line and red line) is 2,280 s (viz. 38 minutes).

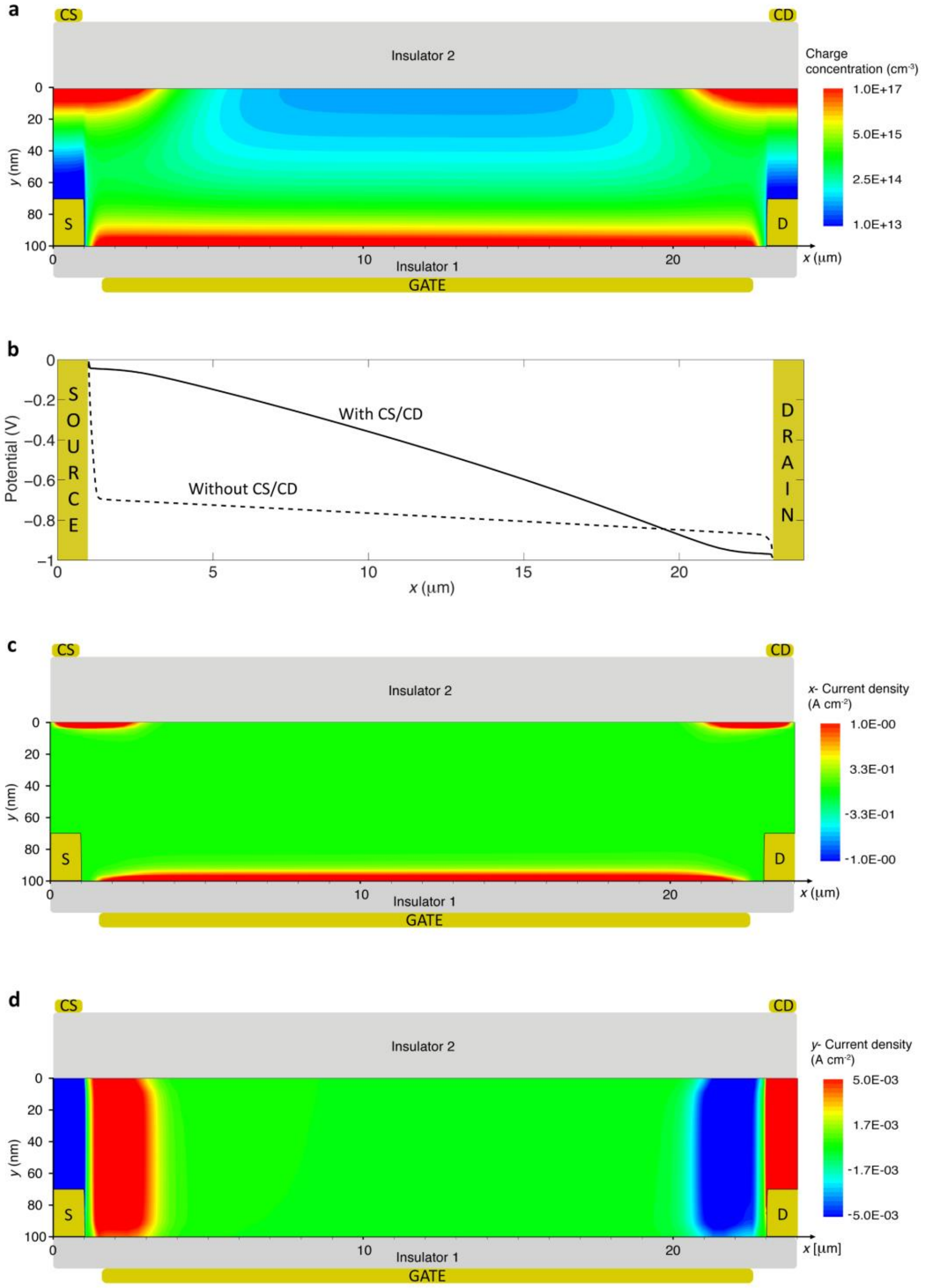


**Supplementary Figure 3 | 2D numerical simulations of the DOFET without gate-source/drain overlap.** The distance between source and drain is 22 µm and the gate length is 20 µm. The gate is not overlapped with the source, drain, control source and control drain electrodes because the charge carriers are injected by the edge of the accumulated CS and CD regions. The CS and CD electrodes are overlapped only with S and D, respectively. The applied voltages are $V_G$ = -5.1 V, $V_S$ = 0 V, $V_D$ = -1 V, $V_{CS}$ = -60 V, $V_{CD}$ = -60 V. For the sake of clarity, the positions of control source (CS), control drain (CD) and gate electrodes are shown. Geometrical and physical parameters are listed in Supplementary Fig. 1 and in the Method section, respectively. (**a**) DOFET cross-section: charge concentration in the organic semiconductor. (**b**) Quasi-Fermi potential at $y$ = 99 nm with (full line) and without (dashed line) CS/CD. Without CS/CD about 80% of $V_{DS}$ drops at the contacts. (**c**) Current density: x-component $J_X$. (**d**) Current density: y-component $J_Y$. The spatial distribution of the $J_X$ current flowing in the transistor channel confirms that the charge carriers are injected/extracted to/from the channel by the CS/CD regions and not by the S/D electrodes as in conventional transistors.

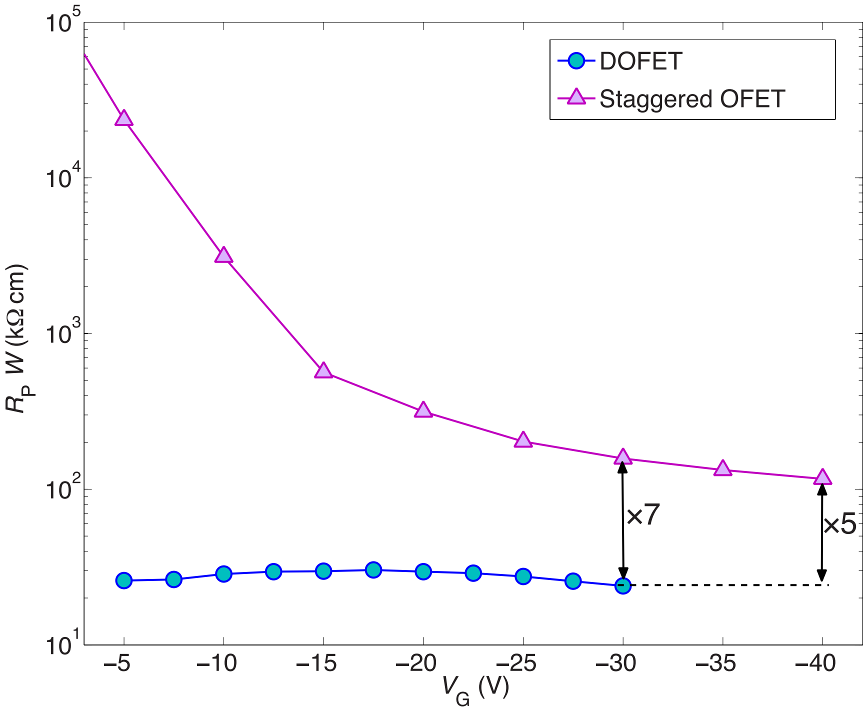


**Supplementary Figure 4 | Contact resistance of the DOFET and of the conventional staggered OFET.** Width-normalized contact resistance $R_P$ as a function of the gate voltage $V_G$. $R_P$ is calculated according to [1]. The applied voltages are $V_S$ = 0 V, $V_D$ = -1 V, $V_{CS}$ = -40 V, $V_{CD}$ = 0 V. The DOFET and the conventional staggered OFET geometries are the same: $W$ = 100 µm, $L$ = 12.5 µm. Further geometrical details are shown in Supplementary Figs. 1b and 1c. As expected, the contact resistance of the staggered OFET is $V_G$ dependent: the source and drain contact resistances get lower by increasing the gate voltage. In contrast, in the DOFET the contact resistance is independent of $V_G$ and it is always much smaller than that of the conventional staggered OFET. In the DOFET the residual contact resistance can be attributed to the drain side, where the contact resistance is not enhanced ($V_{CD}$ = 0 V). It is worth noting that the gate electric-field in the staggered OFET biased at $V_G$ = -40 V ($E_{Y\text{-}G(STAG)}$ = 1.14 MV cm$^{-1}$) is the same of that at the control source in the DOFET ($E_{Y\text{-}CS}$ = 1.14 MV cm$^{-1}$ ) because the insulator is the same (viz. insulator 2, Supplementary Figs. 1b and 1c).

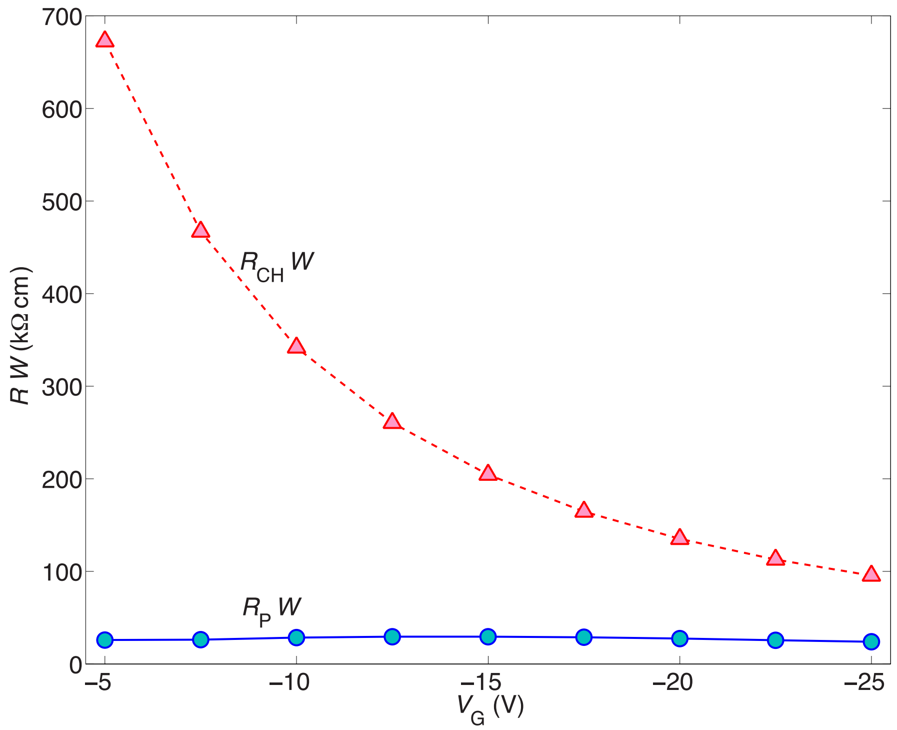


**Supplementary Figure 5 | Contact resistance and channel resistance of the DOFET.** Width-normalized contact $R_P$ and channel $R_{CH}$ resistances as a function of the gate voltage $V_G$. The applied volatages are $V_S$ = 0 V, $V_{CD}$ = 0 V, and $V_{CS}$ = -20 V. The channel width and length are $W$ = 100 µm, and length $L$ = 12.5 µm, respectively. All the geometries are shown in Supplementary Fig. 1b. $R_P$ and $R_{CH}$ are calculated from the measurements with the method [1]. $R_P << R_{CH}$, and, thanks to the charge diffusion, $R_P$ is independent of the gate voltage. It is worth noting that the "residual" contact resistance $R_P$ can be further reduced because the measured $R_P$ = 20 kΩ cm is obtained when the CD region is not formed ($V_{CD}$ = 0 V), and thus, an energy barrier is still present at the drain.

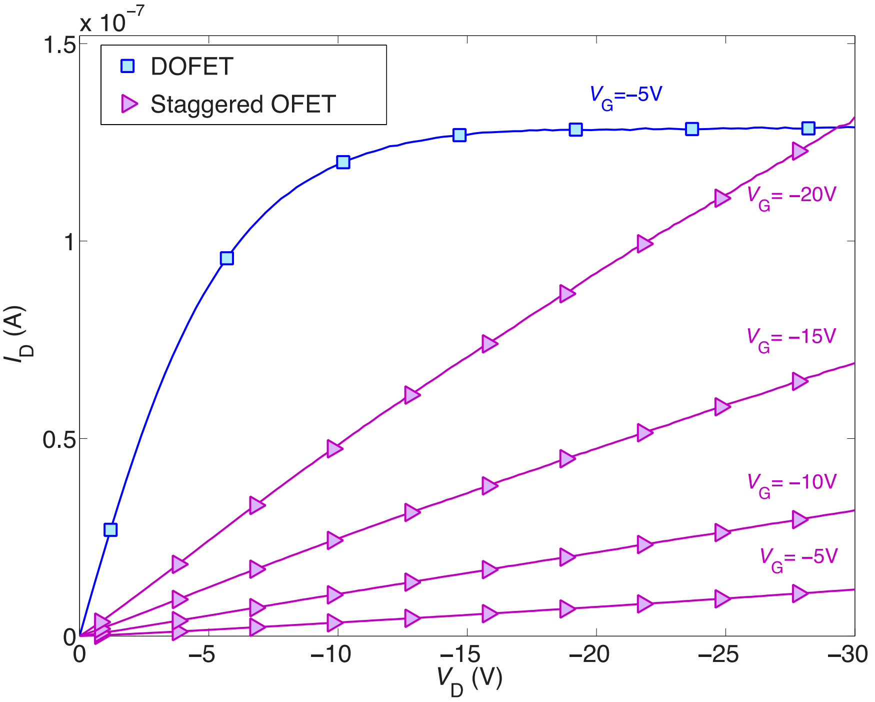


**Supplementary Figure 6 | Output characteristics of the DOFET and of the conventional staggered OFET.** Measured $I_D$-$V_D$ of the DOFET and staggered OFET. The applied voltages are $V_S$ = 0 V, $V_{CS}$ = 0 V, $V_{CD}$ = -60 V. The DOFET and the staggered OFET geometries are the same: $W$ = 100 μm, $L$ = 12.5 μm. Further geometrical details are shown in Supplementary Figs. 1b and 1c. In a staggered transistor the channel-length modulation is large because the source and drain electrodes are at the opposite side of the gate: the electric-field-lines spread from the drain electrode in the semiconductor inducing a large depletion area before they reach the gate electrode. Therefore, the carriers should cross a large resistive zone before reaching the drain. The width of the depleted region depends on the drain voltage eventually leading to a variation of the channel length. This effect is inherently due to the transistor architecture. On the contrary, in a DOFET the channel-length modulation is suppressed and it bahaves like an ideal current generator.

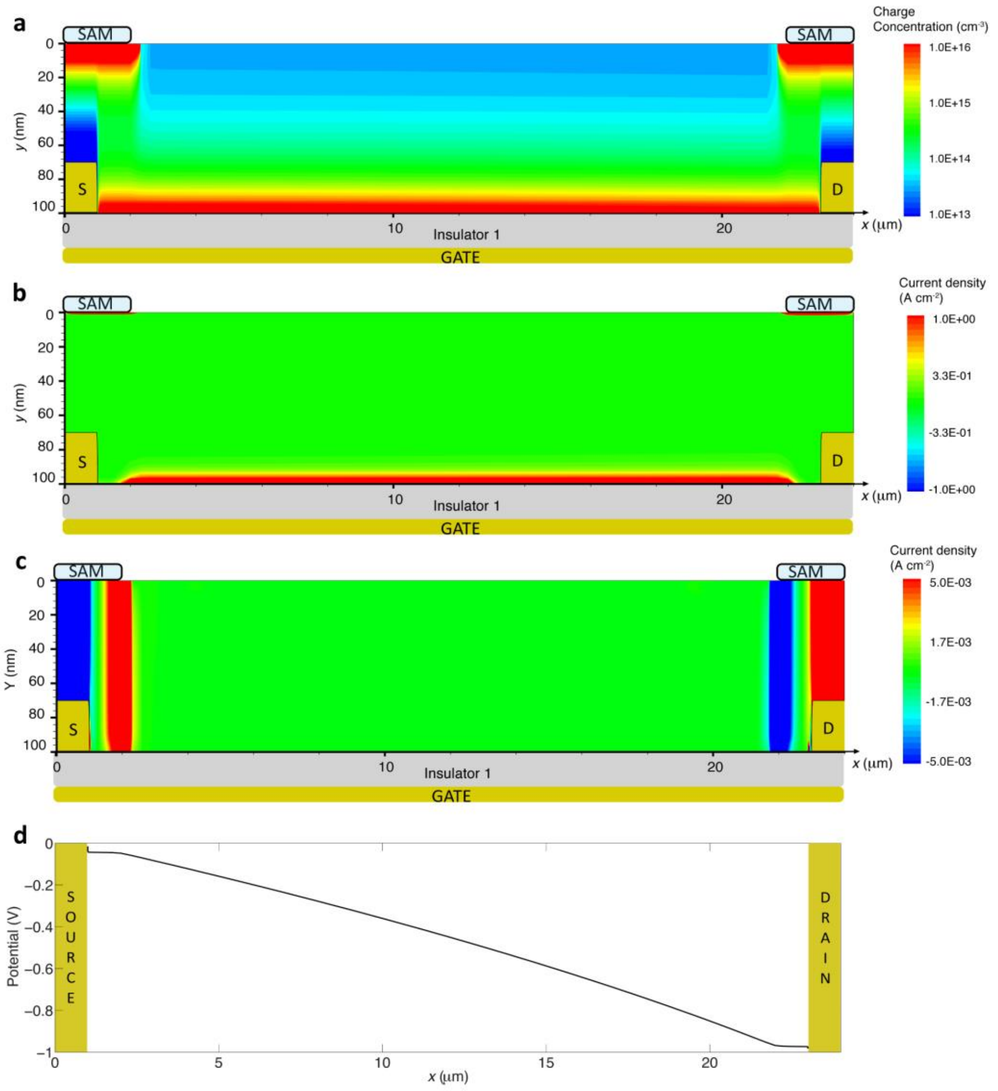


**Supplementary Figure 7 | 2D numerical simulations of the DOFET architecture with SAM.** The applied voltages are $V_G$ = -5.1 V, $V_S$ = 0 V, $V_D$ = -1 V. Geometrical and physical parameters are listed in Supplementary Fig. 1 and in the Method section, respectively. According with [2], the height of the SAM is 1.3 nm and the SAM-induced carrier density is $10^{12}$ $cm^{-2}$. (**a**) Charge concentration in the organic semiconductor. (**b**) Current density: x-component $J_X$. The spatial distribution of the $J_X$ current flowing in the transistor channel confirms that the charge carriers are injected/extracted to/from the channel by the CS/CD regions and not by the S/D electrodes as in conventional transistors. (**c**) Current density: y-component $J_Y$. (**d**) Quasi-Fermi potential at $y$ = 99 nm. The voltage drop at the contacts is negligible.

## Supplementary References

[1] Torricelli, F., Ghittorelli, M., Colalongo, L. & Kovacs-Vajna, Z. M. Single-transistor method for the extraction of the contact and channel resistances in organic field-effect transistors. *Appl. Phys. Lett.* **104,** 093303 (2014).

[2] Calhoun, M. F., Sanchez, J., Olaya, D., Gershenson, M. E. & Podzorov, V. Electronic functionalization of the surface of organic semiconductors with self-assembled monolayers, *Nat. Mater.* **7,** 84-89 (2008).
